# A case of ANCA-associated vasculitis in a 16-year-old female following SARS-COV-2 infection and a systematic review of the literature

**DOI:** 10.1186/s12969-022-00727-1

**Published:** 2022-08-13

**Authors:** Maria C. Bryant, L. Terry Spencer, Ali Yalcindag

**Affiliations:** 1 Department of Pediatrics Hasbro Children’s Hospital Warren Alpert Medical School Brown University, 02903 Providence, RI USA; 2 Division of Pediatric Pulmonology, Department of Pediatrics Hasbro Children’s Hospital Warren Alpert Medical School Brown University, 02903 Providence, RI USA; 3 Division of Pediatric Rheumatology, Department of Pediatrics Hasbro Children’s Hospital Warren Alpert Medical School Brown University, 02903 Providence, RI USA

## Abstract

**Background:**

Antineutrophil cytoplasmic antibody (ANCA)-associated vasculitis (AAV) is a rare form of vasculitis in children. SARS-CoV-2, the virus that causes COVID-19 infection, seems to trigger autoimmunity and new-onset autoimmune disease in pediatric and adult patients. We present a case of new-onset AAV following COVID-19 infection in an adolescent patient, and we review the literature of AAV following COVID-19 infection.

**Case presentation:**

An adolescent female with a history of asthma was diagnosed with mild COVID-19 infection and subsequently developed persistent cough, wheezing, hearing loss, arthralgias, and rash. Her imaging and laboratory workup showed pulmonary nodules and cavitary lesions, elevated inflammatory markers, negative infectious testing, and positive ANCA. She was treated with glucocorticoids, rituximab, and mycophenolate mofetil. At six-month follow-up, she had improvement in her symptoms, pulmonary function tests, imaging findings, and laboratory markers.

**Conclusions:**

We report the second case of new-onset anti-PR3, C-ANCA vasculitis and the fourth case of pediatric-onset AAV following COVID-19 infection. A systematic review of the literature found 6 cases of new-onset AAV in adults after COVID-19 infection. Pediatric and adult patients who develop AAV post COVID-19 infection have few, if any, comorbidities, and show marked radiographic and symptomatic improvement after treatment. There is increasing evidence for COVID-19-induced autoimmunity in children and our case highlights the importance of considering AAV in a child following a recent COVID-19 infection because timely treatment may improve clinical outcomes.

## Background

Antineutrophil cytoplasmic antibody (ANCA)-associated vasculitis (AAV) is a rare form of vasculitis in children with an estimated annual incidence of < 1 per one million children [1–4]. Granulomatosis with polyangiitis (GPA), one of the ANCA-associated vasculitides, is a systemic, necrotizing vasculitis with granulomatous inflammation that typically affects the upper and lower respiratory tract and kidneys [5]. It often presents with nonspecific symptoms including fever, malaise, weight loss, anorexia, myalgias and arthralgias. Although the mechanism of pathogenesis is not fully understood, AAV is thought to be immune-mediated with a chronic and relapsing course [6].

Coronavirus disease 2019 (COVID-19) caused by severe acute respiratory syndrome coronavirus 2 (SARS-CoV-2) has caused significant morbidity and mortality since it was first reported in late 2019 [7]. SARS-CoV-2, similar to other viruses, seems to trigger autoimmunity in both the pediatric and adult populations [8–11]. Acute COVID-19 infection causes less severe symptoms in the pediatric population [12, 13]; however, a small percentage of children subsequently develop immune-mediated disease after COVID-19 infection [8, 9, 14]. We present a case of new onset AAV, most consistent with GPA, following COVID-19 infection in a 16-year-old female, and we review the literature of AAV following COVID-19 infection to highlight another potential SARS-CoV-2 triggered immune-mediated disease in the pediatric population.

## Case presentation

A 16-year-old female with a past medical history significant for asthma was referred to the pediatric pulmonology clinic by her pediatrician for persistent cough and wheezing following COVID-19 infection. Her COVID-19 infection consisted of mild upper respiratory symptoms with anosmia and was diagnosed via PCR.

 Approximately 1 week after recovering from COVID-19, she developed wheezing and a prominent, non-productive cough. She was initially treated by her pediatrician with albuterol which helped her wheezing but not her cough. A chest x-ray at that time was normal. Over the next month, her symptoms progressed to include sinus pain, serosanguinous ear drainage, and a sensation of fullness in her ears. Her pediatrician treated her with courses of azithromycin, cefdinir, and doxycycline. The antibiotics did not resolve her symptoms and she was trialed on a 5-day course of prednisone (60 mg daily). She saw otolaryngology who diagnosed her with chronic bilateral serous otitis media and recommended tympanostomy tube placement. She underwent tympanostomy tube placement with some relief in her symptoms but reported ongoing bilateral hearing loss. She had a CT scan following tympanostomy tube placement that showed bilateral opacified mastoid air cells consistent with chronic inflammation.

When she presented to pediatric pulmonology 6 weeks after the onset of her symptoms, she was still wheezing and had a daily paroxysmal cough with occasional post-tussive emesis. She also reported chest tightness and difficulty breathing. She had a chest x-ray which showed patchy airspace disease of the upper lungs, concerning for a multifocal infectious or inflammatory process. Her symptoms suggested an exacerbation of asthma for which she was prescribed another 5-day course of prednisone (40 mg twice daily), albuterol, and she was started on inhaled corticosteroids in combination with a long-acting beta agonist. Her symptoms improved initially but returned as soon as she stopped taking systemic corticosteroids. Over the next 5 weeks, the patient’s cough became productive of green sputum with worsening wheezing that was no longer responsive to bronchodilators. She was treated with a 28-day course of cefdinir for protracted bacterial bronchitis. When she returned for follow up three months later, she continued to complain of cough and wheezing with new onset myalgias. She underwent pulmonary function testing which showed moderate post-bronchodilation small airway obstruction.

**Fig. 1:**
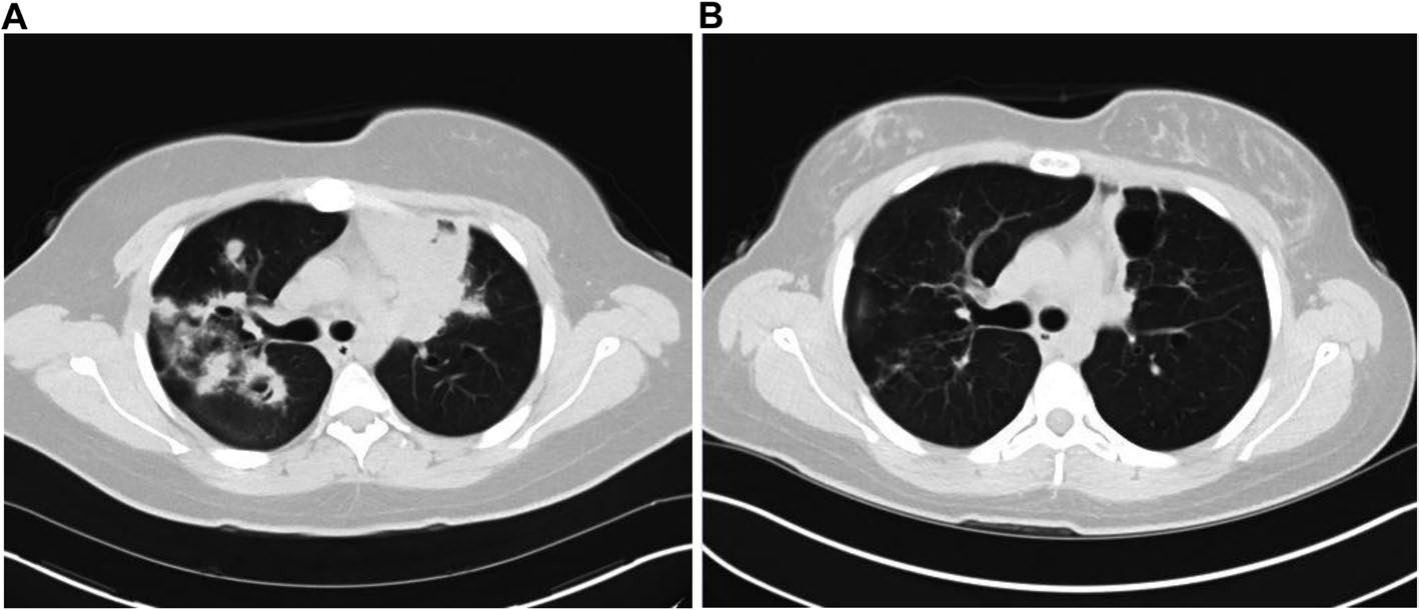
**A** High resolution CT chest taken four months after the onset of symptoms shows extensive multifocal pulmonary nodules and regions of consolidation with areas of cavitation and central bronchiectasis. **B** Repeat high resolution CT chest taken 7 months after initial imaging (**A**) shows an overall improvement in the extent of previously seen multifocal consolidation, nodularity, and cavitation in the lungs, with scattered regions of scarring and several persistent but much smaller nodules. Patient had successfully completed systemic corticosteroid and rituximab treatment at the time of imaging

Chest x-ray revealed perihilar and bilateral upper lobe consolidations that represented a significant progression from her previous x-ray 3 months prior. High resolution chest CT demonstrated extensive multifocal pulmonary nodules and regions of consolidation with multiple areas of cavitation and central bronchiectasis with diffuse bronchial wall thickening as well as reactive mediastinal and hilar adenopathy (Fig. 1A). CT findings were concerning for allergic bronchopulmonary aspergillosis or chronic pulmonary aspergillosis. However, the patient underwent aspergillus antibody studies which were negative. (1–3) Beta-D-Glucan (Fungitell) assay was negative. No IFN-gamma response to M tuberculosis antigens was detected (Quantiferon assay). Induced sputum culture was negative for bacteria, fungal organisms, and acid-fast bacilli. Other laboratory parameters were notable for elevated inflammatory markers with a C-reactive protein of 12 mg/L (0.0–10.0 mg/L) and an erythrocyte sedimentation rate of 30 mm/h (0.0–20.0 mm/h). Her ANCA screen was positive: her C-ANCA was positive with a titer of 1:40 (< 1:20). P-ANCA was negative. Proteinase 3 antibody (anti-PR3) was positive with an antibody index (AI) of 1.4 (0.0–0.9 AI). Antinuclear antibody (ANA) screen was positive with a titer of 1:40 in a speckled pattern. Anticardiolipin antibodies, myeloperoxidase antibodies and anti-glomerular basement membrane antibodies were negative. Beta 2 Glycoprotein 1 IgM and IgG were negative; Complement C3 was 165 mg/dL (88–193 mg/dL) and Complement C4 was 23 mg/dL (15–57 mg/dL). A complete blood count (CBC) was notable for a white blood cell count (WBC) of 11.3 × 10^9/L (3.5–11.0 × 10^9/L), hemoglobin of 13.5 g/dL (11.4–15.4 g/dL), hematocrit of 41.6% (34.2–46.2%), platelet count of 464 × 10^9/L (150–400 × 10^9/L). Serum creatinine and urinalysis were normal. Initial pulmonary function testing revealed a moderate obstructive pattern. She was referred to pediatric rheumatology for concern of systemic vasculitis.

When she presented to pediatric rheumatology clinic, her review of systems was noteworthy for a 45-pound weight loss; intermittent conjunctivitis; sinus congestion, purulent nasal discharge, bilateral hearing loss, ear drainage, sinus headaches, recurrent nosebleeds; cough, wheezing and shortness of breath; progressively worsening bilateral arthralgias in her knees, feet and elbows that improved with activity; intermittent rashes, and sun sensitivity.

She underwent lung tissue biopsy which showed non-specific findings with focal areas of parenchymal atelectasis as well as scattered intra-alveolar macrophages. There was no evidence of acute or granulomatous inflammation or vasculitis identified in the biopsied specimen. There were no yeast or fungal elements seen.

Our patient’s clinical presentation and positive serology is most consistent with GPA. To induce remission, she was started on rituximab infusions which she tolerated well. She completed a glucocorticoid taper and was started on mycophenolate mofetil as maintenance therapy. At her most recent follow up, she reported a marked improvement in her symptoms although she continues to have bilateral conductive hearing loss necessitating hearing aids. Repeat CT imaging showed an overall improvement in the extent of previously seen multifocal consolidation, nodularity, and cavitation in her lungs (Fig. 1B). She demonstrated significant improvement in her pulmonary function tests (Table 1), and her ANCA and PR3 antibodies have remained negative to date.

**Table 1:** Pulmonary function tests before and after AAV treatment

Pulmonary function tests prior to treatment			Pulmonary function tests after treatment	
		% Predicted		% Predicted
FVC (L)	2.62	75	3.88	96
FEV1 (L)	1.95	63	3.04	86
FEV1/FVC (%)	74.6	85	68.0	78
FEF 25–75% (L/Second)	1.82	1.82	2.80	68
PEF (L/Second)	2.27	30	5.15	73

*FEV1* Forced expiratory volume in one second, *FVC, *Forced vital capacity *FEV1/FVC ratio, * Percentage of the FVC expired in one second *FEF* 25–75%,  Forced expiratory flow over the middle one-half of the FVC *PEF, * Peak expiratory flow

## Discussion

Here we report the second case of new-onset anti-PR3, C-ANCA vasculitis and the fourth case of AAV following COVID-19 infection in the pediatric population. The gold standard for diagnosing childhood vasculitis is with either histopathology from tissue biopsy or by characteristic lesions detected by imaging studies. Multiple studies in adults with AAV have found that less than half of all biopsies show the characteristic inflammatory and vasculitic changes to confirm the diagnosis [15–17], and sampling difficulty is often a limitation which could have explained our patient’s biopsy findings. Different classification criteria have been proposed to diagnose GPA in children [5, 18–20]. The mostly commonly used classification system in pediatrics is the European League Against Rheumatism/Pediatric Rheumatology International Trials Organization/Pediatric Rheumatology European Society (EULAR/PRINTO/PRES) classification scheme; it requires three of the following six features to diagnose GPA: abnormal urinalysis with hematuria or proteinuria; upper airway involvement (chronic purulent or bloody nasal discharge, nasal septum perforation or saddle nose deformity, chronic or recurrent sinus inflammation); granulomatous inflammation on biopsy; subglottic, tracheal or endobronchial stenosis; abnormal chest x-ray or CT; ANCA positivity (MPO-ANCA, PR3-ANCA or no specificity) [5, 18, 21]. Most patients (> 80%) have a positive ANCA, and GPA is primarily associated with PR3-ANCA [22, 23]. Approximately 90% of patients with GPA have ear, nose and throat (ENT) manifestations such as rhinosinusitis, otitis media, earache, conductive and/or sensorineural hearing loss, persistent rhinorrhea, purulent or bloody nasal discharge [24–26]. Our patient meets criteria for AAV and her clinical presentation—upper airway and middle ear involvement with saddle-nose deformity, recurrent nosebleeds, bilateral chronic serous otitis media and conductive hearing loss, abnormal CT showing cavitary lesions, and PR3 and C-ANCA positivity—is most consistent with GPA [18].

## Search strategy and literature review

The authors conducted a systematic review of the literature from December 1, 2019 to January 1, 2022, in PubMed/MEDLINE, combining the MeSH search terms (COVID-19) AND (ANCA-associated vasculitis), filtered by age (birth-18) and date (Jan. 1, 2019-Jan. 1, 2022); ((Adolescent) AND (COVID-19) AND (ANCA-associated vasculitis)); ((Pediatric) AND (COVID-19) AND (ANCA-associated vasculitis)). Two authors (MCB, AY) independently screened titles, abstracts, and full texts of all relevant articles. All articles reporting ANCA-associated vasculitis after COVID-19 infection in the pediatric population were included. From each article, the authors collected data on publication year, age, gender, comorbidities, chronicity with COVID-19 infection, laboratory tests, serological profile, lung pathology, kidney pathology, AAV therapy, and outcomes.

## Results from the literature review

A review of the literature found three cases of PAAV following COVID-19 infection, and, with the present report, four cases were included in this review [27–29]. Of the four patients (including ours), two were male [27–29]. Two patients had no prior comorbidities while two had pre-existing asthma [29]. Immunological tests showed C-ANCA positivity in two cases [27] and P-ANCA positivity in two [28, 29]. Two patients had anti-PR3 antibodies [27], and two patients had anti-MPO antibodies [28, 29]. Pulmonary imaging studies showed multifocal cavitary pulmonary nodules in two patients [27]; diffuse alveolar hemorrhage in two patients [28, 29]; dense patchy infiltrates in two patients [28, 29]. Two patients had normal kidney function at the time of presentation [27] while two patients had necrotizing glomerulonephritis on renal biopsy [28, 29]. Neither required hemodialysis [30–33]. All four patients were treated with glucocorticoids which are the standard of care for chronic vasculitis in children [34, 35]. Three patients received rituximab therapy [27, 28] and one patient received plasmapheresis [29]; for maintenance therapy, two were treated with cyclophosphamide [28, 29], one was treated with mycophenolate mofetil, and one did not continue therapy [27]. All four patients had symptomatic and radiographic improvement at follow up (Table 2) [27–29].

**Table 2:** Summary of clinical findings, demographics, and treatment strategies of pediatric-onset AAV after COVID-19 infection

	Bryant et al	Powell et al	Fireizen et al	Reiff et al
Age, years	16	12	17	17
Sex	Female	Female	Male	Male
Comorbidities	Asthma	None	Asthma and Obesity	None
Chronology with COVID-19	1–2 weeks following infection	2 weeks following infection	2 months following infection	Concurrent
Positive serology	Anti-PR3 and C-ANCA	Anti-MPO and ANCA	Anti-MPO and P-ANCA	Anti-PR3 and C-ANCA
Lung involvement at presentation	Extensive multifocal pulmonary nodules and regions of consolidation with multiple areas of cavitation and central bronchiectasis with diffuse bronchial wall thickening	Dense consolidation in the left lower lobe and patchy infiltrate in the right middle and upper lobes without ground-glass opacities; diffuse alveolar hemorrhage	Extensive heterogeneous infiltrates in both lungs with an unusual fluffy central distribution concerning for diffuse alveolar hemorrhage	Multiple bilateral cavitary lung lesions
Kidney involvement at presentation	Normal kidney function (Cr. 0.79 mg/dL)	Pauci-immune necrotizing and crescentic glomerulonephritis	Renal biopsy showed necrotizing glomerulonephritis with limited immune complex deposition	Normal kidney function (Cr. 0.74 mg/dL)
AAV treatment	Prednisone, rituximab, mycophenolate mofetil	Methylprednisolone, rituximab, cyclophosphamide	Methylprednisolone, plasmapheresis, cyclophosphamide	Methylprednisolone, rituximab, not on maintenance therapy
Antibody titers at presentation	1:40 and a proteinase 3 antibody (PR3) level of 1.4 (normal < 1.0)	1:640 and a perinuclear pattern	Not available	1:640 and a proteinase 3 antibody (PR3) level of 251.9 (normal < 1.0)
Outcomes	Marked improvement of multifocal consolidation, nodularity, and cavitation on CT	Improvement in clinical symptoms	Resolution of DAH and AKI, not requiring outpatient dialysis	Clinically asymptomatic with marked improvement of cavitary lung nodules on CT

There have been six reported cases of adults developing AAV following COVID-19 infection and a table summarizing these cases is well documented in an article by Izci Duran et al. [30] Similar to our findings, half of the adult patients had C-ANCA vasculitis with anti-PR3 antibodies. The most common lung findings were bilateral cavitary lesions, pulmonary infiltrates, and alveolar hemorrhage which were similar to the findings in children. Kidney involvement was more severe in the adults who developed AAV [30–33]. All adult patients had either crescentic or necrotizing glomerulonephritis on renal biopsy, active urinary sediment, and high creatinine levels. Two patients had kidney failure that required hemodialysis. Studies from the NIH report that glomerulonephritis is present in only 18% of patients at presentation [24]. However, 77 to 85% of patients subsequently develop glomerulonephritis, usually within the first two years of disease onset [24, 36].

The treatment strategy was similar in adults and children with all patients receiving systemic corticosteroids [27–33]. The adult patients received either rituximab or cyclophosphamide for induction therapy [30]. Cyclophosphamide is often added for patients with more severe disease [37]. Other immunosuppressive medications, such as methotrexate and azathioprine, as well as biologic agents (e.g., TNF-inhibitors, rituximab, and tocilizumab) have been used to treat adults with AAV and are increasingly being used to treat chronic vasculitis in the pediatric population [35, 38].

The clinical outcomes following treatment were generally favorable in both adult and pediatric patients: all patients had symptomatic and radiographic improvement; the patients with renal involvement had improvement or resolution of their hematuria, proteinuria and creatinine levels at follow-up; none required long-term dialysis; Two patients (one child and one adult) had bilateral hearing loss as part of their initial presentation [30]. Unfortunately, both continued to have profound hearing loss with the need for hearing aids or cochlear implants [30].

## Conclusion

Our case presentation and review of the pediatric and adult literature show that AAV may be another autoimmune-mediated sequela of COVID-19. We report the tenth case of AAV following COVID-19 infection. In general, patients who develop AAV after COVID-19 infection have few, if any, comorbidities, and show marked radiographic and symptomatic improvement after treatment.

Autoimmunity may be generated by a combination of genetic, hormonal, and environmental factors in susceptible people [39, 40]. One such environmental trigger is viral illness. Studies have suggested a causal relationship between viral infections and the onset of autoimmune disease with molecular mimicry, hyperstimulation, dysregulation of the immune system, and complement activation being proposed mechanisms [10, 41]. Epstein-Barr virus, cytomegalovirus, and human immunodeficiency virus are examples of viruses that have an established association to multiple autoimmune diseases [42–44]. There is increasing evidence that SARS-CoV-2 is another virus that can lead to dysregulation of the immune system and the development of autoimmune disease in children and adults [11]. A recent study found that children with a history of COVID-19 infection were at an increased risk of developing type 1 diabetes mellitus than those without a history of COVID-19 [9]. Antecedent viral infections have been implicated in the development of PAAV including GPA [45]. Our report and review of the literature suggest that COVID-19 may be another viral trigger for the development of AAV in children and adults. One proposed mechanism for the development of autoantibodies including ANCA in the context of COVID-19 infection is the presence of extensive neutrophil infiltration at sites of tissue necrosis and immunothrombosis that could contribute to tissue tolerance failure with antibody production [46]. More research is needed to better understand how SARS-CoV-2 may act as a precipitating trigger of autoimmunity and autoimmune disease in children. Our case highlights the importance of considering AAV in a pediatric patient presenting with pulmonary and renal disease following a recent COVID-19 infection because timely treatment may improve clinical outcomes [45].

## Data Availability

All data regarding this study has been reported in the manuscript. Please contact the corresponding author if you are interested in any further information.

## Abbreviations

ANCA: Antineutrophil cytoplasmic antibody
AAV: ANCA-associated vasculitis
COVID-19: Coronavirus disease 2019
GPA: Granulomatosis with polyangiitis
PAAV: Pediatric-onset ANCA-associated vasculitis
SARS-CoV-2: Severe acute respiratory syndrome coronavirus 2
